# Co-occurrences of fall-related factors in adults aged 60 to 85 years in the United States National Health and Nutrition Examination Survey

**DOI:** 10.1371/journal.pone.0277406

**Published:** 2022-11-08

**Authors:** Shirley Rietdyk, Satyajit Ambike, Steve Amireault, Jeffrey M. Haddad, Guang Lin, David Newton, Elizabeth A. Richards

**Affiliations:** 1 Department of Health and Kinesiology, Purdue University, West Lafayette, Indiana, United States of America; 2 Center on Aging and the Life Course, Purdue University, West Lafayette, Indiana, United States of America; 3 Data Science Consulting Service, Purdue University, West Lafayette, Indiana, United States of America; 4 Department of Mathematics, Purdue University, West Lafayette, Indiana, United States of America; 5 Department of Statistics, Purdue University, West Lafayette, Indiana, United States of America; 6 Department of Mechanical Engineering, Purdue University, West Lafayette, Indiana, United States of America; 7 School of Nursing, Purdue University, West Lafayette, Indiana, United States of America; University of Illinois at Chicago, UNITED STATES

## Abstract

A broad set of factors are associated with falling (e.g., age, sex, physical activity, vision, health), but their co-occurrence is understudied. Our objectives were to quantify the *number* and *pattern* of co-occurring fall-related factors. Data were obtained from the U.S. National Health and Nutrition Examination Survey (N = 1,957, 60–85 years). Twenty fall-related factors were included (based on previous research), covering a wide range including cognitive, motor, sensory, health, and physical activity measures. The number and pattern of co-occurring fall-related factors were quantified with logistic regression and cluster analyses, respectively. Most participants (59%) had ≥4 fall-risk factors, and each additional risk factor increased the odds of reporting difficulty with falling by 1.28. The identified clusters included: (1) healthy, (2) cognitive and sensory impaired, and (3) health impaired. The mean number of co-occurring fall-related factors was 3.7, 3.8, and 7.2, for clusters 1, 2, and 3, respectively (p<0.001). These observations indicate that co-occurrence of multiple fall-risk factors was common in this national sample of U.S. older adults and the factors tended to aggregate into distinct clusters. The findings support the protective effect of physical activity on fall-risk, the association between gait speed and falls, and the detrimental effect of health-related factors on difficulty with falls (e.g., arthritis, prescription medications). Cluster analyses revealed a complex interplay between sex and BMI that may alter the role of BMI in the etiology of falls. Cluster analyses also revealed a large detrimental effect of health-related factors in cluster 3; it is important to extend current fall interventions (typically focused on balance, flexibility, strength, cognitive, fear factors) to include health-related interventions that target factors such as BMI and arthritis.

## 1. Introduction

Falls and fall-related injuries are substantial public health problems, resulting in significant personal and economic burdens. In 2018, 27.5% of older adults in the U.S. fell and 10.2% sustained a fall-related injury [1]. Globally, falls are a leading cause of fatal and non-fatal health loss [2]. Injuries from falls lead to chronic pain, functional impairment, loss of independence, and reduced quality of life [3, 4]. Even if a person is not injured, fear of falling can limit social and physical activities, leading to social isolation, cognitive decline, depression, physical deconditioning, and reduced quality of life [5–7]. The economic burden related to falls was $50 billion for medical costs in 2015 [8]. Costs associated with hospitalization, readmission, and post-acute care for traumatic injury substantially exceeded those costs due to congestive heart failure, pneumonia, stroke, and acute myocardial infarction [9].

Many factors are associated with falls, including but not limited to age, sex, ethnicity/race, physical inactivity, gait abnormality, muscle weakness, sensory impairment, cognitive impairment, medication use, history of falls, pain, and diseases such as arthritis, cardiovascular disease, cancer, and diabetes [10–18]. The co-occurrence of multiple diseases and medical conditions (i.e., multimorbidities, [19]) is common [20]. Similarly, multiple fall-risk factors co-occur in individuals [10–18]. The relationship between number of risk factors and falls is additive [18]; it is important to extend this research to also quantify the *number* of co-occurring risk factors present in a population and the *patterns* of co-occurring risk factors. Cluster analysis can identify how these factors aggregate in order to better understand fall-risk in individuals, rather than focusing on average values for representative populations [10].

Multiple studies have demonstrated the feasibility and utility of cluster analysis in understanding fall risk [10–17]. One set of these studies limited the *examined factors* to either (1) chronic disease/multi-morbidity [11, 17], or (2) health behaviors (smoking, alcohol consumption, physical activity) [13]. Extending cluster analyses to include a more comprehensive set of fall-risk factors is essential given that falls are a complex, multi-system problem (e.g., [10]). Another set of cluster analysis studies limited the *examined population* to specific groups (patients admitted to hospital for falls or were outpatients) [14–16]. To our knowledge, only one study with cluster analysis has included a comprehensive set of fall-risk factors (e.g., chronic disease, medications, physical and cognitive impairments, health factors) with a population-based cohort study (Swedish adults) [12]. This study indicated that the risk factors aggregated in specific patterns and the clusters demonstrated different levels of fall-risk [12]. The identified clusters emphasized the importance of a multifactorial perspective and the need for a holistic approach when reducing fall risk. For example, while the association between increasing age and fall-risk is well known [1, 21], all of the clusters included people from most age groups, highlighting the importance of considering the aggregation of factors versus a single factor [12]. Since falls are a growing global burden [22], and patterns of risk factors have been rarely studied [12], it is important to conduct cluster analyses in multiple national population samples to increase knowledge regarding the number and patterns of risk factors. Our objective was to quantify the *number* and *pattern* of co-occurring fall-related factors in a representative U.S. population.

## 2. Methods

### 2.1 NHANES database 1999–2000 and 2001–2002

The National Health and Nutrition Examination Survey (NHANES) is conducted by the National Center for Health Statistics (NCHS) and uses a complex, multistage probability design to obtain a representative, cross-sectional sample of noninstitutionalized United States civilians [23]; NHANES data files are public-use. The NHANES procedures are approved by the NCHS Ethics Review Board. All participants provided their informed consent. Furthermore, NHANES datasets are publicly available and devoid of any individual-level information. Therefore, no further institutional review board approval was necessary.

The survey is a continuous program with a changing focus on various measurements to meet emerging needs. The years 1999–2000 and 2001–2002 included a wide range of key fall-related factors in the following categories: cognitive, motor, sensory, health, and physical activity, which allowed us to achieve our study goals. We identified 24 fall-related factors in the NHANES dataset based on previous research [10–18], but four of these variables had more than 30% missing values. The four variables with missing data included: quadriceps peak force, do you need special equipment for walking (yes/no), general health condition (excellent, very good, good, fair, poor), and chronic obstructive pulmonary disease (yes/no). Since the clustering method we used requires that there is no missing data, including these variables would have substantively reduced the sample size. Therefore, these four variables were removed from the dataset, resulting in 20 fall-related factors (Table 1).

**Table 1:** Means and proportions of the fall-related factors, expressed for all data combined, and for each cluster. Numeric scores are reported as means, categorical scores are proportions (prop.), as indicated with each variable. P-values are Benjamini-Hochberg adjusted (allows for a 5% false discovery rate). Post hoc analyses to compare clusters were Bonferroni adjusted (*p* = 0.0167); significant post hocs are noted with an asterisk for each cluster comparison (cluster 1 versus 2 = 1 v 2, etc.). ‘Likert’ indicates Likert scale from 1–4 with 1 = no difficulty and 4 = unable.

Variable	All data (N = 1957) mean (SE)	Cluster 1 (N = 911) mean (SE)	Cluster 2 (N = 754) mean (SE)	Cluster 3 (N = 292) mean (SE)	1 v 2	1 v 3	2 v 3	*p* value
*Demographic Variables*								
Age	70.49 (0.17)	66.22 (0.17)	74.90 (0.25)	72.52 (0.47)	*	*	*	<0.001
Race (prop. white)	0.62 (.001)	0.64 (0.02)	0.58 (0.02)	0.64 (0.03)				0.0855
Sex (prop. male)	0.49 (0.01)	0.45 (0.02)	0.61 (0.02)	0.34 (0.03)	*	*	*	<0.001
*Health Related Variables*								
Arthritis (prop. yes)	0.44 (0.01)	0.37 (0.01)	0.42 (0.02)	0.74 (0.03)		*	*	<0.001
Heart Failure (prop. yes)	0.05 (0.00)	0.02 (0.00)	0.05 (0.01)	0.11 (0.02)	*	*	*	<0.001
Cancer (prop. yes)	0.18 (0.01)	0.16 (0.01)	0.21 (0.01)	0.20 (0.02)	*			0.0357
Stroke (prop. yes)	0.05 (0.00)	0.02 (0.00)	0.07 (0.01)	0.07 (0.02)	*	*		<0.001
Number Prescription Med.	2.61 (0.05)	2.24 (0.07)	2.48 (0.08)	4.16 (0.18)		*	*	<0.001
BMI	28.07 (0.11)	28.41 (0.16)	26.56 (0.16)	30.94 (0.40)	*	*	*	<0.001
*Self-report Limitations/Difficulty Variables*								
Assistive Device (prop. yes)	0.02 (0.00)	0.00 (0.00)	0.01 (0.00)	0.08 (0.02)		*	*	<0.001
Limitations from Work (prop. yes)	0.07 (0.01)	0.04 (0.01)	0.06 (0.01)	0.16 (0.02)		*	*	<0.001
Difficulty Walk ¼ Mile (Likert)	1.34 (0.01)	1.02 (0.01)	1.14 (0.01)	2.66 (0.05)		*	*	<0.001
Difficulty Walk Up 10 Step (Likert)	1.25 (0.01)	1.08 (0.01)	1.08 (0.01)	2.23 (0.05)		*	*	<0.001
Difficulty Stand from Chair (Likert)	1.18 (0.01)	1.05 (0.01)	1.08 (0.01)	1.82 (0.05)		*	*	<0.001
*Sensory*, *Cognitive*, *Balance*, *and Gait Variables*								
Visual Acuity (denominator)	40.97 (0.84)	31.14 (0.68)	50.66 (1.67)	46.83 (2.65)	*	*	*	<0.001
Cognitive Function	44.32 (0.41)	54.41 (0.52)	34.58 (0.55)	37.79 (1.03)	*	*	*	<0.001
Balance Time to Failure (s)	41.82 (0.85)	62.77 (1.06)	21.25 (1.16)	29.16 (2.04)	*	*	*	<0.001
20 ft Walk Test (s)	6.81 (0.06)	5.88 (0.04)	7.18 (0.07)	8.82 (0.29)	*	*	*	<0.001
*Physical Activity (PA) Variables*								
PA—Household (min/week)	123.51 (6.10)	156.75 (9.96)	112.05 (9.54)	48.27 (8.31)	*	*	*	<0.001
PA—Leisure time, moderate & vigorous (min/week)	154.38 (5.9)	202.89 (9.34)	135.00 (9.73)	51.47 (7.78)	*	*	*	<0.001

### 2.2 Study population

**Fig 1:**
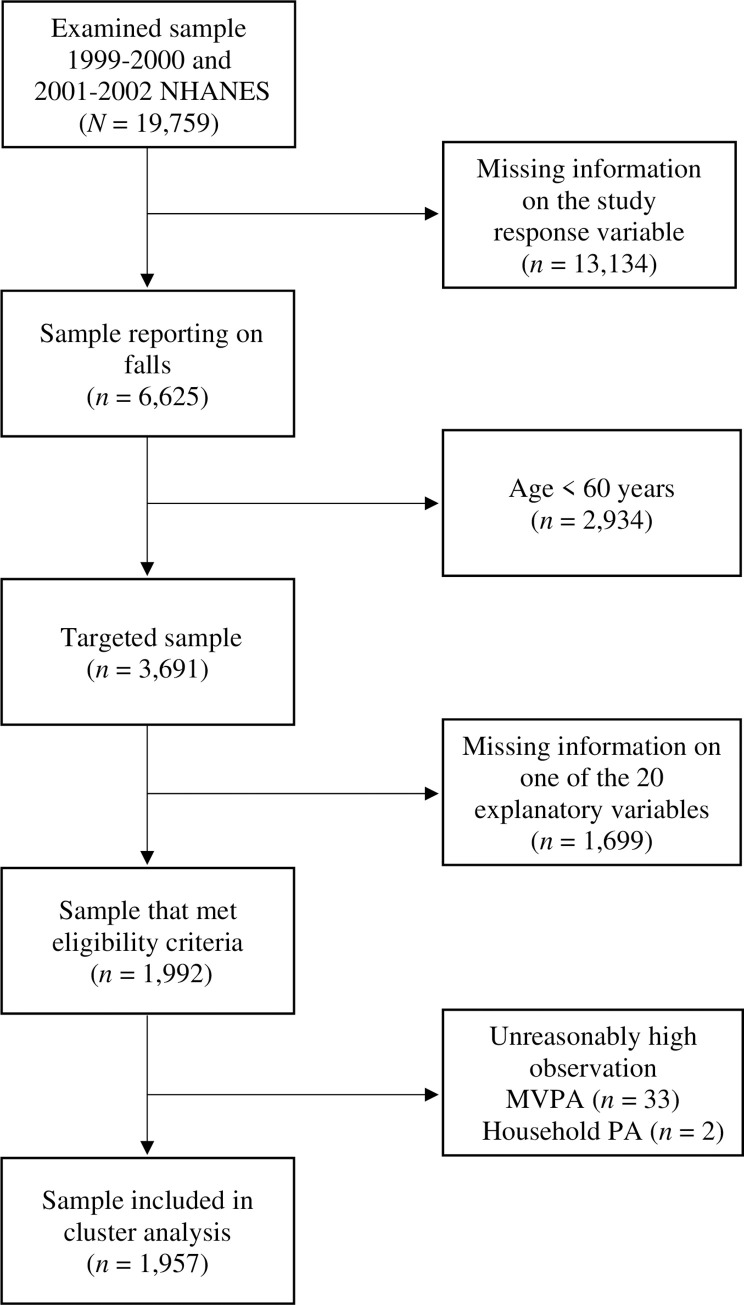
Participants flow diagram. MVPA: Moderate-to-vigorous physical activity. PA: physical activity.

Inclusion criteria was being aged 60 to 85 years. Exclusion criteria was missing variables in the selected variables (as cluster analysis requires a complete dataset). The NHANES database for 1999–2000 and 2001–2002 includes 3,691participants representing the U.S. population aged 60 to 85 years (when all ages are included: 19,759 participants). After participants with missing variables were excluded, there were 1,992 participants. In addition, 35 participants reported unreasonably high levels of physical activity and were removed from the analytical sample (details provided in Section 2.5). Therefore, we included and analyzed data on 1,957 participants (aged 60–85 years) (Fig 1).

### 2.3 Fall-related factors

The NHANES procedures include in-home interviews and physical examinations conducted in mobile centers. Where the measures are relatively standard (e.g. BMI), the methods below are brief. Where the measures are not as standardized (e.g. Romberg test of standing balance), the methods provide more detail. Readers can find detailed descriptions of the NHANES data collection techniques at the website (http://www.cdc.gov/nchs/nhanes.htm).

#### 2.3.1 Demographic variables

Age (continuous; years), sex, and race-ethnicity (dichotomized into white or non-white).

#### 2.3.2 Health-related variables

presence of chronic disease (arthritis, congestive heart failure, history of stroke, history of cancer; dichotomized as yes/no), body mass index (BMI) (continuous), number of prescription medications (integer), and use of assistive device (none, cane, walker; dichotomized into yes/no).

#### 2.3.3 Romberg test of standing balance

The Romberg test assessed the time (continuous; seconds) for which participants could stand on firm and foam surfaces, with eyes open and eyes closed [24]. The balance testing quantified the duration a participant could stand unassisted under four conditions: 1) firm surface, eyes open, 2) firm surface, eyes closed, 3) foam surface, eyes open and 4) foam surface, eyes closed. Test failure was defined as participants opening their eyes during the eyes-closed conditions, moving their extremities to maintain balance, beginning to fall, or requiring assistance to maintain balance. If participants were successful, they moved on to the next condition. Participants were allowed two trials for each condition (15-second tests for conditions 1 and 2 and 30-second tests for conditions 3 and 4). Time to failure was recorded for each test, and the four times were summed, with a maximum of 90 s (completed all four tests successfully). A lower score indicates greater impairment. The Romberg test is reliable [25], and the validity is supported by the ability of the summed score to discriminate fallers from non-fallers [26].

#### 2.3.4 20-foot walk test/gait speed

Time (continuous; seconds) to walk 20 feet (6.1 m) was assessed in all eligible participants aged 50 years and older. Since the distance of 20 feet is constant, the time to walk will also reflect differences in gait speed (speed = distance/time), and in the discussion, the 20-foot walk test is described as gait speed to be consistent with studies that have examined the relationship between gait speed and falls [27–29]. The 6 m walk test has excellent test-retest reliability [30].

#### 2.3.5 Self-report functional limitations

During the interview protocol, all participants were asked if they had physical, mental, or emotional limitations preventing them from working or if they were limited in any way physically, mentally, or emotionally (yes/no). Participants were also asked three questions designed to measure their functional status. Walking (1/4 mile), climbing 10 steps, and standing up (from an armless chair) difficulty were assessed on a Likert-scale, ranging from 1 (*no difficulty*) to 4 (*unable*) (integer). Reliability of the functional limitations is excellent, and the validity is supported by correlations with age, exercise frequency, and death [31].

#### 2.3.6 Cognitive function

During the in-home interview, the Digit Symbol Substitution Test (DSST) was used to assess the cognitive function of participants over 60 years of age. Participants were asked to draw as many symbols as possible that were paired with numbers within two minutes. The score is the number of correct symbols drawn in two minutes; one point for each correctly drawn and matched symbol, and one point subtracted for each incorrectly drawn and matched symbol, with a maximum score of 133 (integer) [32]. Sample items were provided for initial practice. The DSST provides a valid and sensitive measure of cognitive function (Jaeger 2018) [33].

#### 2.3.7 Physical activity

During the in-home interview, all participants were given a physical activity questionnaire that assessed participation in household and leisure-time physical activity (separately) during a typical week. Weekly minutes of household, moderate-intensity, and vigorous-intensity physical activity were calculated based on self-reported number of days and minutes per day participating in activity of each intensity (continuous; minutes). Previous research indicates that participants tend to overestimate their physical activity with self-report, but the self-report measure is highly correlated with objective physical activity measures [34]. Thus, self-report physical activity can be used to accurately rank people within a sample [34].

#### 2.3.8 Visual acuity

Measures of visual acuity were taken in both eyes. Similar to [35], the two visual scores were analyzed as follows: (1) vision worse than 20/200 was changed to 20/200, (2) the highest visual acuity in both eyes was selected as the single visual acuity score, (3) for those with visual acuity in only one eye, this was selected as the single visual acuity score.

### 2.4 Falls variable

Note this variable was not used as a fall-related factor (i.e. the 20 variables listed above), but rather to see if the number and pattern of fall-related factors were related to participant’s self-report of difficulty with falling.

#### 2.4.1 Difficulty with falling

Because the number of falls was not collected concurrently during the years with the other variables of interest (e.g., cognitive scores, balance, and gait assessments) in NHANES, we used the following question from the survey as a proxy for falls: “During the past 12 months, have you had dizziness, difficulty with balance, or difficulty with falling?” (dichotomized as yes/no; hereafter “difficulty with falling”). In the 1,957 participants included here, 506 (26%) replied ‘yes’. The falls variable would ideally be number of falls, but number of falls was not collected concurrently with the other variables of interest (e.g., cognitive scores, balance, and gait assessments) in NHANES. However, we expect that the variable “difficulty with falling” will be correlated with falls; this expectation is supported by the observation that 26% of participants reported difficulty with falling, which is similar to the 27.5–29% of older adults reporting falls in the literature [1, 36].

### 2.5 Data analysis

The NHANES sample was filtered to exclude anyone aged less than 60 years (Fig 1). The clustering methods we used require that there is no missing data, so any participants with missing data were excluded (Fig 1). We investigated each of the numerical variables for outliers using histograms and the IQR and z-score methods. Thirty-three participants reported unreasonably high moderate to vigorous leisure-time physical activity (over 1,260 minutes/week, which translates to over three hours/day), and two participants reported over seven hours/week of household physical activity; these 35 participants were identified as univariate outliers and were removed from the analytical sample. For the remaining variables, there were a few suggested outliers, but the values were not extreme and did not merit removal based on the values alone. Moreover, we checked the sensitivity of each suspected outlier by running our analysis with and without each data point, and ensured that the removal/inclusion did not affect any of the conclusions. As outlined below, parametric tests included the Wald-test for the individual logistic regression models, and Chi-square tests, one-way ANOVA F-tests, two-sample proportion z-tests, and two-sample t-tests for analyzing the differences across clusters. Each of these parametric tests are robust to deviations in most of their key assumptions with a large sample size (such as the NHANES dataset included here). Data cleaning and preprocessing were done using Python 3.7 and R (Version: 3.6.1). Statistical analysis was performed using R.

#### 2.5.1 Bivariate analyses

Logistic regression (Wald-test) was performed on each of the 20 fall-related factors individually, in order to identify the relationship of each fall-related factor with reporting difficulty with falling. P-values from the Wald-tests were adjusted with Benjamini-Hochberg (BH) procedure to account for multiple tests, allowing for at most a 5% false discovery rate. Odds ratios and corresponding confidence intervals were computed.

#### 2.5.2 Study objective 1: Quantify the number of co-occurring fall-related factors

To quantify the number of co-occurring fall-related factors, for each participant, we computed the total number of risk factors that were identified in the bivariate analysis as associated with difficulty with falling. For example, an individual with two risk factors could include being female and having cancer. For numeric variables, if the participant had a score that was above the 75^th^ percentile for the data reported in the sample here, it was considered a risk factor. The maximum number of risk factors that a participant could have with this sample was 17, as 17 of the 20 fall-related factors were significantly associated with difficulty with falling in the bivariate analysis (described in the Results section). A logistic regression model was used to estimate the (multiplicative) increase in odds of having difficulty with falling for every additional risk factor.

#### 2.5.3 Study objective 2: Quantify the pattern of co-occurring fall-related factors

K-prototypes clustering [37] was performed using the clustMixType R package [38] to uncover latent sub-groups, or clusters, within the dataset. The dataset included all 20 fall-related factors but did not include the measure ‘difficulty with falling’. The dataset included numeric and categorical variables. Therefore, K-prototypes partitional clustering algorithm was utilized, which is capable of handling both types of variables [37]. K-prototype uses different distance metrics for numeric and categorical data (Euclidean distance for numeric; Hamming distance for categorical), with a coefficient γ that controls the relative weight between the categorical and numeric variables. The γ coefficient was set to 1. This value reflects an unbiased choice since both types of variables are weighted equally. The clustering algorithm was run with different choices of cluster numbers (from two to eight). Within-cluster variance was plotted as a function of the number of clusters to generate an elbow plot. Based on visual inspection of the elbow plot, the number of clusters was set to three so that a reasonable amount of variance was explained with a parsimonious model.

As noted in the methods, 35 participants were excluded due to unreasonably high reports of physical activity (Fig 1). We conducted the cluster analysis with and without these 35 participants, and of the 1957 participants, 15 people changed clusters. Since the inclusion/exclusion of the 35 participants had a limited effect on the results, and since their physical activity values were unreasonable, we chose to report results obtained without including these participants.

*2*.*5*.*3*.*1 Fall-related factors across the clusters*. Each fall-related factor was examined to determine if it was statistically different across the three clusters, and thereby contributed to the formation of the clusters. Chi-square tests were used for each categorical variable to detect significant associations with cluster labels. Similarly, one-way ANOVA tests were used for the numeric variables. P-values from these tests were adjusted using the BH procedure to control the false discovery rate at 0.05. Two-sample proportion z-tests were computed for the categorical variables (proportions), and two-sample t-tests were used for the numeric variables (means). Post hoc analyses with Bonferroni adjustments (*p* = 0.0167) were performed to compare cluster means (or proportions) for each fall-related factor.

*2*.*5*.*3*.*2 Number of co-occurring fall-related factors across the clusters*. The number of co-occurring fall-related factors was examined with one-way ANOVA tests to determine the difference across the three clusters. Two-sample t-tests were used for post hoc analyses with Bonferroni adjustments (*p* = 0.0167) to compare cluster means.

*2*.*5*.*3*.*3 Effect of the cluster on the likelihood of reporting difficulty with falling*. Chi-square tests were used to determine if the likelihood of reporting difficulty with falling was significantly different across the three clusters. We tested to see if the proportions were significantly different for each pair of clusters with a two-sample proportion z-test. The confidence interval for each proportion was computed using standard 1-sample-z proportion confidence intervals (CI); the CIs were then Bonferonni-corrected to take into account multiple intervals (*p* = 0.0167).

### 3. Results

Demographic information and the fall-related factors for the sample are listed in Table 1.

### 3.1 Bivariate analyses

**Fig 2:**
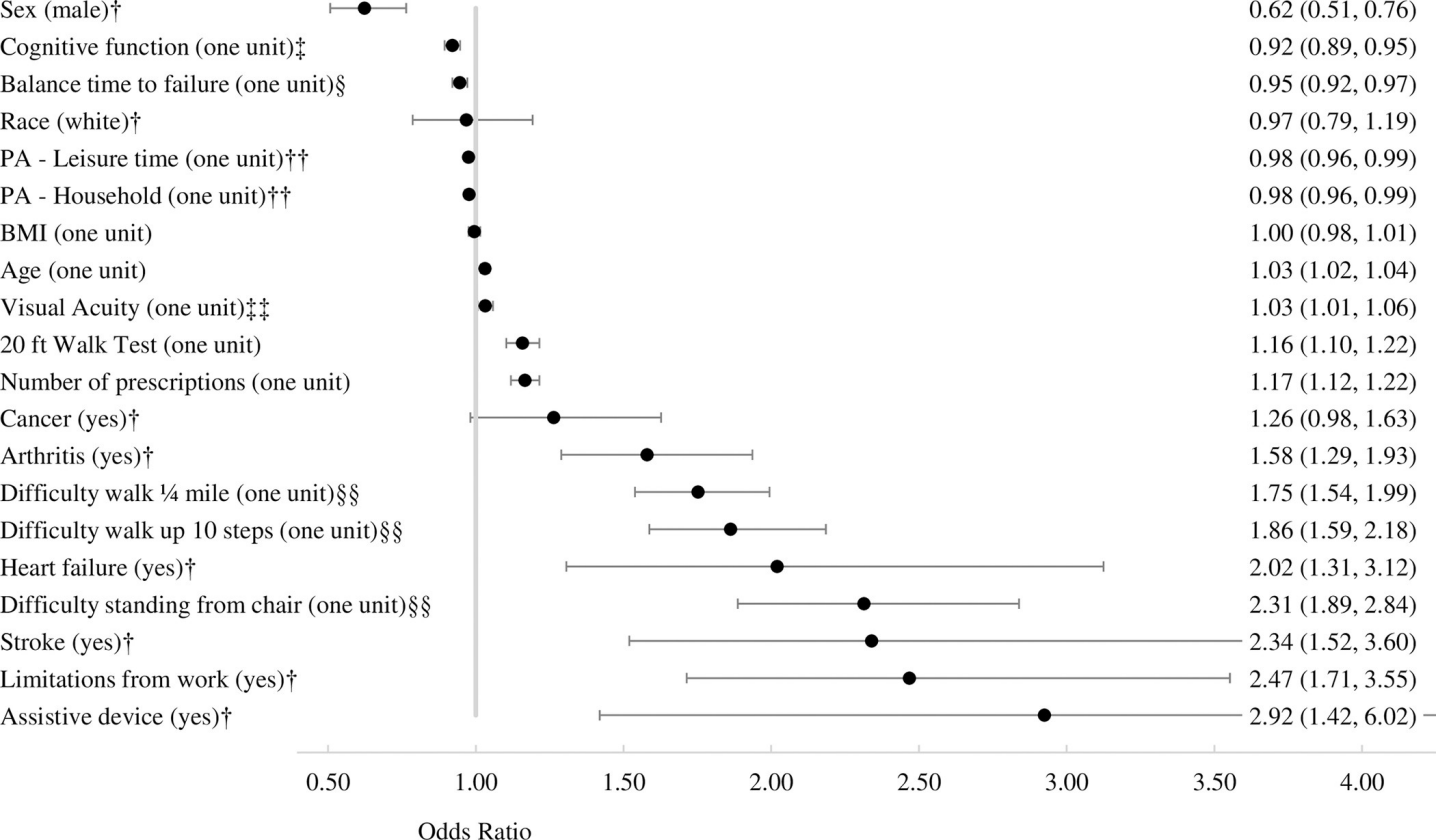
Bivariate associations between the 20 fall-related factors (listed on the left) and reporting difficulty with falling (N = 1,957). A logistic regression (and corresponding Wald-test) was conducted between each fall-related factor and reporting difficulty with falling. Odds ratio (95% Wald confidence intervals). All odds ratios were significant (p≤0.02) with the following exceptions: race, BMI, and cancer (p≥0.08). PA = physical activity. † dichotomous variables. For continuous and integer variables, each unit corresponds to the unit of measurement (e.g., one unit of age is one year, one unit of 20 ft walk test is one second), with the following exceptions: ‡ for cognitive function, one unit is five points on Digit Symbol Substitution Test (max score 133). § for balance, one unit is 10 s during the Romberg test of standing balance (max score 90 s). †† for PA, one unit is 30 minutes of PA. ‡‡ for visual acuity, one unit is 10 ft in the denominator of the visual acuity score (e.g., 20/20 to 20/30). §§ Likert scale 1–4, one unit is one integer on the scale.

Seventeen of 20 variables had a significant association with the reporting of difficulty with falling (Fig 2, *p* < 0.02). The three exceptions were cancer (*p* = 0.08), BMI (*p* = 0.66), and race (*p* = 0.76*)*. The results of the bivariate analysis (Fig 2) are consistent with published research on falls [39–43], supporting our use of reporting difficulty with falling as a proxy for falls for the NHANES dataset.

### 3.2 Study objective 1: Number of co-occurring fall-related factors

**Fig 3:**
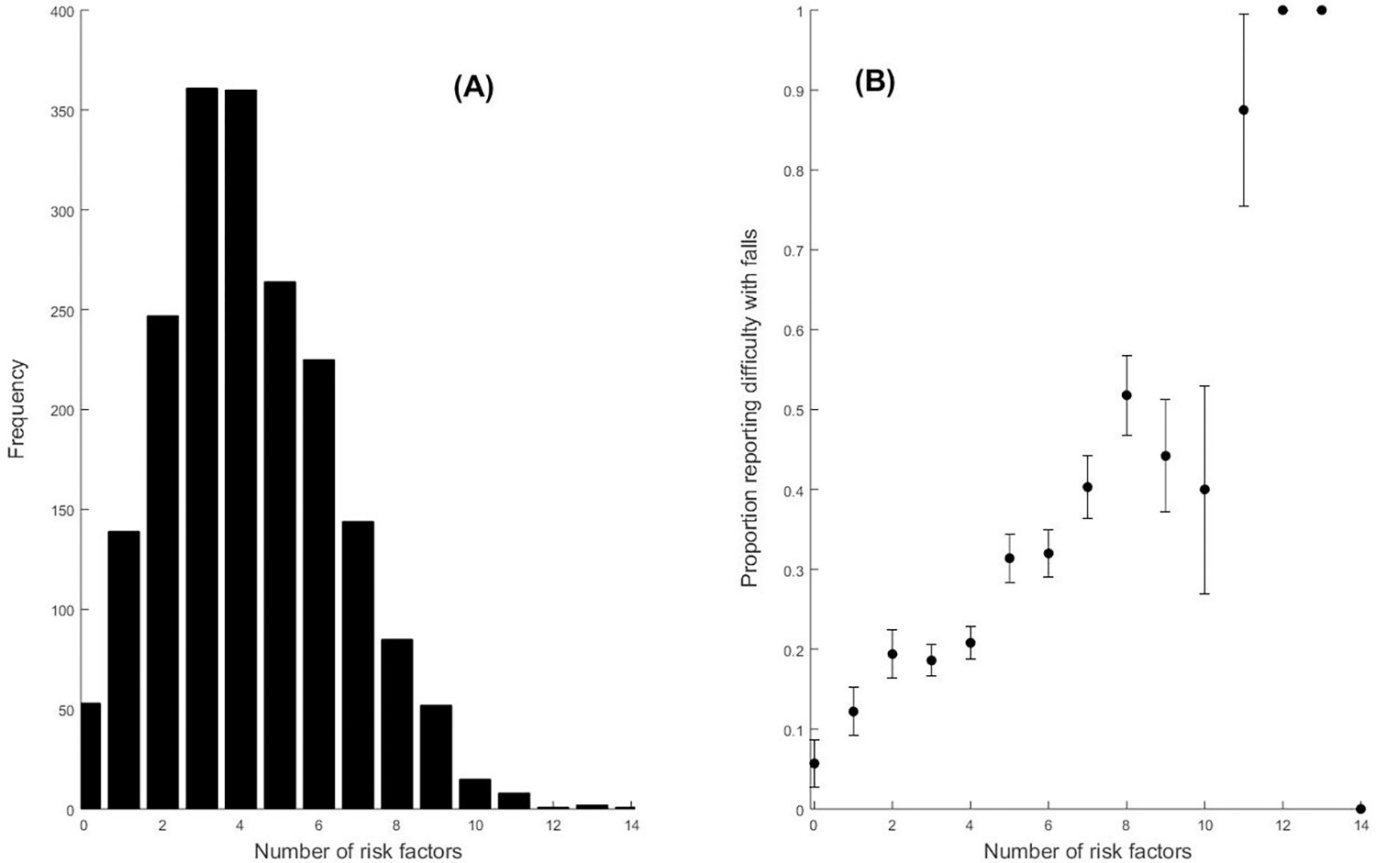
(A) Frequency histogram for the number of fall-related factors for all participants (N = 1,957). (B) Proportion of participants reporting difficulty with falling as a function of the number of fall-related factors. Error bars indicate Bonferroni-adjusted confidence intervals; confidence intervals were not calculated when the number of risk factors was greater than 11 due to the low numbers of participants in those categories.

The number of risk factors for each participant ranged from 0 to 14 (maximum possible was 17); the majority of the participants (59%) had four or more risk factors, while few (0.6%) had 11 or more risk factors (Fig 3A). With each additional risk factor, we observed a multiplicative increase of 1.28 (1.22, 1.34) in the odds of reporting difficulty with falling (Fig 3B).

### 3.3 Study objective 2: Pattern of co-occurring fall-related factors with cluster analysis

**Fig 4:**
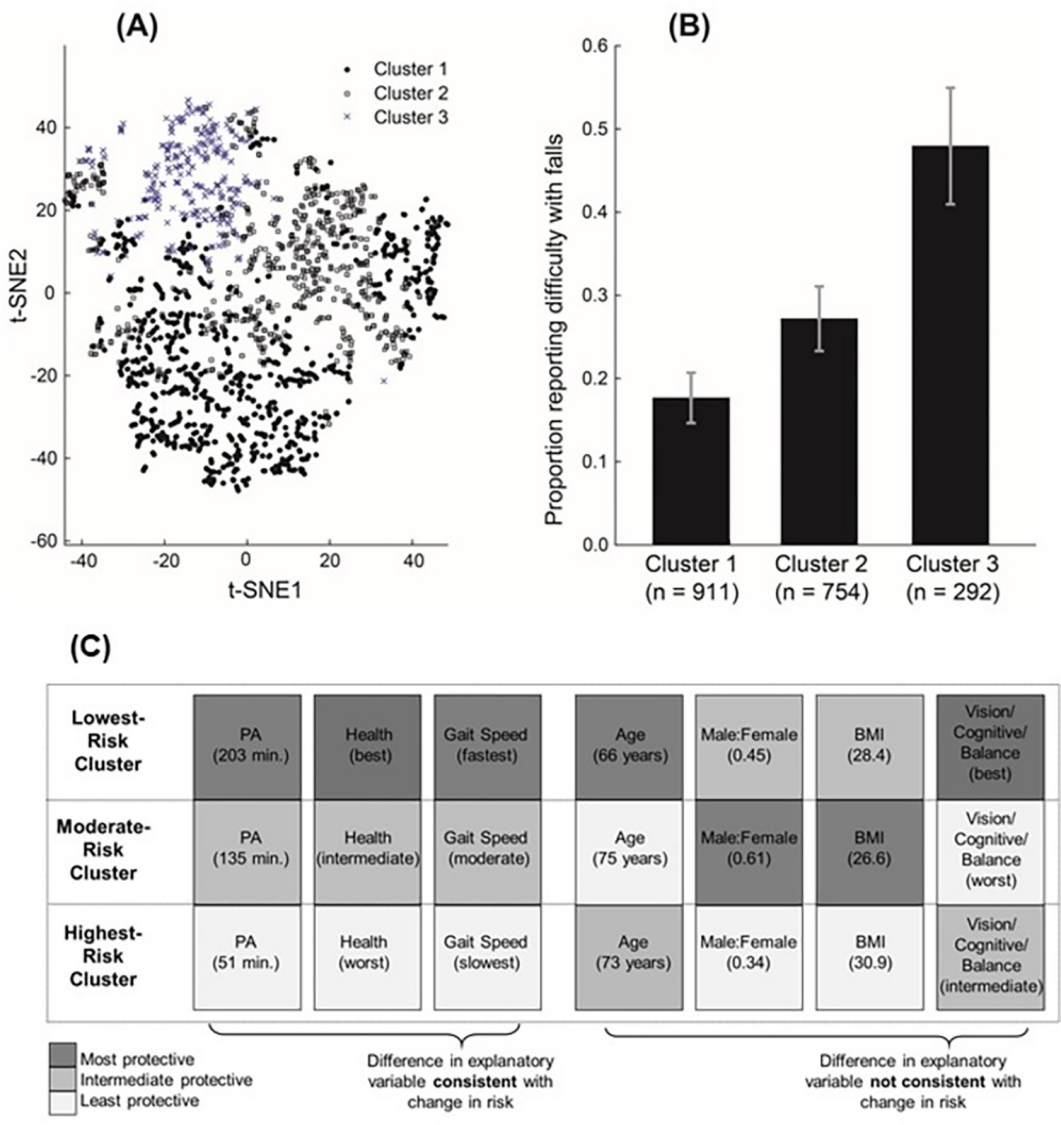
(A) Clustering results with t-distributed stochastic neighbor embedding (t-SNE2) plot (N = 1,957). A t-SNE2 plot embeds high-dimensional data to allow visualization in two-dimensional space. (B) Proportion of people reporting difficulty with falling in each cluster. Error bars indicate Bonferroni-adjusted confidence intervals. (C) Summary of the fall-related factors for the three clusters. Darker boxes indicate variables associated with lower likelihood of reporting difficulty with falling, and vice versa. The first three columns include the fall-related factors that had monotonic differences across clusters that were parallel to the difference in reporting with difficulty with falling. In the remaining four columns, the differences were not parallel. Mean values shown in brackets for physical activity (PA), age, and body mass index (BMI). Male: Female is the ratio of males to females; vision/cognitive/balance is a qualitative combination of the three factors.

The assignment of individuals to three clusters is depicted in Fig 4A. Nineteen of the 20 fall-related factors were significantly different across at least two of the clusters (*p* < 0.04; Table 1). The single exception was race (*p* = 0.086). Post hoc comparisons across the clusters are indicated in Table 1. A heat map of the fall-related factors (Fig 4C) assists in visualizing the pattern of fall-related factors for each cluster. A number of fall-related factors demonstrated a monotonic change across the three clusters, and these are presented in the left columns of Fig 4C. Fall-related factors that were significantly different across the clusters but did not show a monotonic change are presented in the right columns of Fig 4C. The variables associated with vision (visual acuity), cognitive (DSST score), and balance (Romberg test) are presented together (column on far right, Fig 4C) as they demonstrated the same pattern across clusters. Based on the fall-related factors, clusters 1, 2, and 3 are labeled “healthiest”, “cognitive and sensory impaired” and “health impaired”, respectively, as outlined below.

The number of fall-related factors within each cluster was significantly different (*p* < 0.001). The mean number of risk factors were 3.7, 3.8, and 7.2, for clusters 1, 2, and 3, respectively. Post hoc comparisons revealed that the means of clusters 1 and 2 were not significantly different from each other, and cluster 3 had a greater mean number of risk factors than clusters 1 and 2 (*p* < 0.001).

The proportion of participants reporting difficulty with falling was significantly different across clusters (χ^2^ = 106.84, *p* < 0.001, Fig 4B), with significant differences between each pair of clusters and a monotonic increase from Clusters 1 to 2 to 3 (Fig 4B).

#### 3.3.1 Cluster 1: “Healthiest” (*n* = 911, 47% of sample, mean age 66.2 ± 5.1 years (mean ± standard deviation), 55% female)

Cluster 1 included males and females in almost equal numbers, and relative to the other two clusters, had the youngest age, was the least likely to report diseases (therefore labeled ‘healthiest’), had an intermediate BMI (classified as overweight), and had the most protective sensory, motor, and cognitive scores. They were the most physically active, achieving 135% of the physical activity guidelines for Americans (i.e., 150 minutes of moderate to vigorous physical activity a week [44]). Overall, Cluster 1 had scores on the fall-related factors that were almost always more protective relative to the other clusters (Table 1; Fig 4B and 4C). The only fall-related factors that did not have the most protective scores were sex and BMI.

#### 3.3.2 Cluster 2: “Cognitive and sensory impaired” (*n* = 754, 39% of sample, mean age 74.9 ± 6.9 years, 39% female)

Cluster 2 had the least protective scores in the cognitive, sensory, and balance tests (Table 1; Fig 4B and 4C). Therefore, this cluster is labeled “cognitive and sensory impaired”. Cluster 2 had more males, was the oldest group, was intermediate to the other two clusters on health-related factors, and had the lowest BMI (but still classified as overweight). They achieved 90% of the physical activity guidelines for Americans.

#### 3.3.3 Cluster 3: “Health impaired” (*n* = 292, 15% of sample, mean age 72.5 ± 8.0 years, 66% female)

Cluster 3 had the most compromised health (Table 1; Fig 4B and 4C), and was therefore labeled “health impaired”. Cluster 3 had more females, an intermediate age, the highest proportions of participants reporting the presence of disease, and the highest number of prescription medications. In particular, arthritis was present in 74% of the participants, and the BMI of 30.9 kg/m^2^ is categorized as obese. Scores in the sensory, cognitive, and balance tasks were intermediate to the other two clusters. They had the lowest measures of physical activity, achieving 34% of the physical activity guidelines for Americans.

## 4. Discussion

Our objective of this study was to quantify the *number* and *pattern* of co-occurring fall-related factors. The strengths of this study include the wide range of co-occurring fall-related factors, and the large national sample of participants. The majority of the participants (59%) had four or more risk factors. The cluster analysis revealed that the fall-related factors tended to aggregate, resulting in the identification of three distinct clusters, labeled as (1) healthiest, (2) sensory and cognitive impaired, and (3) health impaired. The first two clusters had similar mean numbers of fall-related factors (3.7 and 3.8), while the third cluster had almost double the number of fall-related factors (7.2).

Co-occurrence of multiple fall-related factors was common in this national sample; less than 10% had zero or one risk factor and the majority (59%) had four or more risk factors (Fig 3). These observations support and quantify the contention that fall-risk factors co-occur in individuals [10–17]. The observation that the majority had four or more fall-related factors is especially relevant, as previous research combined all participants with four or more factors into a single group [18]; the data here indicate that each additional risk factor beyond four increased the odds of having difficulty with falling (Fig 3B), so combining them into a single group obscures relevant information. While previous research has demonstrated that the number of risk factors is *additive* for probability of falling [18], here we observed that the risk of having difficulty with falls showed the familiar “S-shaped” logistic curve as a function of the number of risk factors (Fig 3B). Visual examination of Fig 3B indicates that the relationship appears linear until about seven co-occurring factors, which is consistent with the linear relationship in [18] (the scale goes from 0 to 4+ co-occurring factors in [18]), and the non-linear component appears to begin after seven co-occurring factors. The consistency across the two studies (at least up to four co-occurring factors) strengthens the observations, especially given the differences across the two studies. Differences included falls measure: self-report of difficulty with falling vs prospective fall survey, current study vs [18], respectively; number of participants: 1,957 vs 336; years of data collection: 1999–2002 vs 1985, and assessments: both studies included cognitive, motor, and vision factors, but they were assessed differently. While a multiplicative relationship has clinical relevance, future research is required to validate this multiplicative relationship.

The cluster analysis demonstrates that certain fall-related factors tend to co-occur (Fig 4A and 4C), and the patterns indicate that some fall-related factors appear to demonstrate a straightforward relationship with difficulty with falling, while other factors demonstrate a complex interplay between the factors which is not captured by the bivariate analysis. Several variables increased monotonically across the three clusters (physical activity, health-related variables, and gait speed; Fig 4C, Table 1); in a parallel fashion, reporting difficulty with falling also increased monotonically across the three clusters (Fig 4B). These parallel monotonic changes indicate a relatively straightforward relationship between the fall-related factors and reporting difficulty with falling. The inverse relation between physical activity and the proportion of people reporting difficulty with falling demonstrate the protective effects of physical activity on falls [45], and is consistent with the potential dose-response relationship between the amount of physical activity and fall-risk [46]. The positive relation between reporting difficulty with falling and higher numbers of people reporting chronic conditions (arthritis, heart failure, cancer, and stroke), as well as higher number of prescription medications (Table 1), is consistent with the observation that the number of chronic conditions and number of prescription medications are related to falls [17, 47]. The relationship between gait speed and the proportion of people reporting difficulty with falling is also consistent with the association between gait speed and fall-risk [27–29]. Note also that gait speed and health appear associated with each other (Fig 4C), consistent with the association between gait speed and health [48, 49]. We also note that a cluster analysis using a national sample from Sweden [12] had similar observations on physical activity, chronic diseases, and gait speed. The cluster with the highest fall-risk was the most inactive, had the highest proportion of people with two or more chronic diseases, and had the slowest gait. While it is difficult to compare across cluster analyses due to the different fall-related factors selected, and the different number of clusters (three clusters in the current study, five clusters in [12]), the commonalities across the Swedish and American populations strengthen the observations and highlight further the protective effect of physical activity and the strong association between falls, health, and gait speed.

However, most of the fall-related factors did not show monotonic changes across the clusters, including these factors: age, proportion of females, vision, cognitive, balance, BMI (Fig 4C, Table 1). Rather, cluster 2 demonstrated the lowest cognitive and sensory scores, and the oldest age (Fig 4C). Despite these features, cluster 2 reported lower difficulty with falling than cluster 3, likely because the physical activity participation for cluster 2 was higher (90% vs. 34% of recommended guidelines). It is also possible that poor health, observed in cluster 3, had a greater impact on difficulty with falling than sensory and cognitive impairment, especially when coupled with the higher proportion of females and the lowest physical activity participation. In addition, cluster 3 had almost twice as many fall-related factors as the other clusters.

The patterns across the three clusters indicate that there is a complex interplay between BMI, sex, and difficulty with falling. In the healthiest cluster 1, the scores on the fall-related factors were the most protective with two exceptions: (i) roughly equal numbers of males and females, and (ii) BMI values in the intermediate range (Table 1; Fig 4C). These patterns revealed by cluster analysis for sex and BMI are not consistent with generally observed associations, including: (i) females are more likely to fall and sustain fall-related injuries [21, 39, 40, 50], which is also observed in our bivariate analysis (Fig 2), and (ii) individuals with higher BMI and/or larger waist circumference are more likely to fall [51, 52], which was not observed in our bivariate analysis of the NHANES sample (Fig 2). A possible explanation is that the fall variable, problems with falling, is more sensitive to BMI for females than for males. This explanation was developed by examining the two exceptions (sex and BMI) in clusters 2 and 3: BMI was lowest for cluster 2, which contained the highest proportion of males, and BMI was highest for cluster 3, which contained the highest proportion of females (Fig 4C). This explanation is supported by previous research that during midlife overweight and obese mid-life females have an increased fall-risk, unlike mid-life males [53], but this research has not yet been extended to older adults. If falls are more sensitive to BMI for females than for males, it will alter the role of BMI in the etiology of falls. Current research on the association of BMI with falls has been adjusted for sex [51] or stratified by age and sex categories [53], but the statistical interaction of sex and BMI on falling has not been systematically examined. Our research suggests that BMI should be coupled with sex (at least) for predicting fall risk.

The cluster analysis also indicates that the cluster with the worst health scores, Cluster 3 Health Impaired, was 66% female and 74% had arthritis. This description is relevant for identifying appropriate fall-reduction interventions. Most evidence-based exercise interventions to prevent falls (e.g., Stepping On) are multifactorial, and include some combination of exercises to address balance, flexibility, strength, cognitive training (e.g., dual-tasks), and the fear of falling [54]. Our analyses suggest that exercise intervention programs may not be as relevant for people in cluster 3 as they have intermediate vision, cognitive, and balance scores. This is clinically relevant because cluster 3 is mostly female, and most attendees of balance-related intervention programs in North America are female [55, 56]. Older adults with impaired health may be better served by including health-related interventions that target factors such as BMI and arthritis. For example, the Fit and Strong! program is designed for people with arthritis [57]. If an older adult with arthritis is falling, an exercise program for arthritis may be more beneficial than an exercise program for balance.

The strengths of this study are the large national dataset of community-dwelling older adults, the wide range of fall-related factors, and the use of objective in-person testing for several of these factors (e.g., cognitive scores, vision, etc.). There were also several limitations to the study. First, our fall-related variable–reporting difficulty with falling–was taken from the question “During the past 12 months, have you had dizziness, difficulty with balance, or difficulty with falling?”. This question is broader than the number of falls, and it overlaps with the fall-related factor that quantified balance. However, this variable is likely correlated with falls, which is supported by (i) the consistency of our bivariate analysis (Fig 2) with published research on falls [39–43] and (ii) the observation that 26% of participants reported difficulty with falling, which is similar to the 27.5–29% of older adults reporting falls in the literature [1, 36]. Further, we had similar observations on physical activity, chronic diseases, and gait speed as a cluster analysis with a national sample from Sweden [12]. Therefore, we determined that the limitations associated with the fall measure were outweighed by the strengths of the NHANES database. Second, several variables were self-reported (e.g., difficulty getting out of a chair, etc.), but a mix of measured and self-reported factors is common in fall-related research [12, 42]. Third, the selection of fall-related factors to include in the analysis, although based on previous research [10–18], was subjective. Clustering analysis is sensitive to the choices researchers make, including which variables to include or remove. There is a trade-off between the number of variables and the number of participants. For example, we wanted to include quadriceps strength, but this inclusion would have reduced the sample size by nearly 25% due to missing data in the NHANES sample. However, the information related to quadriceps strength is likely implicitly incorporated as several variables were moderately correlated with quadriceps strength (20-foot walk test, Difficulty Walking Up 10 Steps, Difficulty Walking ¼ Mile, and others). Therefore, we don’t expect that inclusion of strength would have substantively altered the results. Fourth, the NHANES sample is cross-sectional and not longitudinal. Nonetheless, studies that are cross-sectional in design can be used to quantify covariations among study variables and are appropriate for testing relational hypotheses. Fifth, the data we examined here is already more than 20 years old, and it is possible that the numbers have changed somewhat in the interval. However, the included years had the most comprehensive set of fall-related factors, and again we felt that having a more comprehensive set of variables outweighed a more recent dataset.

In summary, this study on a national sample of U.S. older adults (aged 60 to 85) demonstrated that co-occurrence of four or more fall-related factors was common, and the factors tended to aggregate into distinct clusters. The findings support previously observed relationships with individual fall-related factors, including the protective effect of physical activity on falls [45], and the association between falls and chronic conditions [17], prescription medications [47], and gait speed [27–29]. We also demonstrated that there is a complex interplay between sex and BMI, which may alter the role of BMI in the etiology of falls. Finally, while many fall interventions target balance, flexibility, strength, cognitive training (e.g., dual-tasks), and fear of falling, it appears that older adults with impaired health may be better served by including health-related interventions that target factors such as BMI and arthritis.
